# The relationship between burnout and self-regulation resources in teachers during COVID-19 pandemic

**DOI:** 10.1192/j.eurpsy.2022.1307

**Published:** 2022-09-01

**Authors:** M. Titova, S. Nakhmedova

**Affiliations:** 1 Lomonosov Moscow State University, Department Of Psychology, Moscow, Russian Federation; 2 Lomonosov Moscow State University, Baku Branch, Baku, Azerbaijan

**Keywords:** COVID-19, burnout, self-regulation resources, hardiness

## Abstract

**Introduction:**

Burnout is understood as a stable professional and personal deformation, which can be typical for teachers (Maslach, Schaufeli, 1993). Multilevel structure of burnout that include physiological, affective-cognitive, and behavioral dimensions was described (Perlman, Hartman, 1998). С. Maslach proposes a three-dimensional burnout model, including emotional exhaustion, depersonalization and reduction of personal achievements (Maslach, 2000; Schaufeli, Enzman, 1998). It is especially important to prevent burnout by effective using of self-regulation resources during the COVID-19 pandemic, when the level of stress increases (Samanta et al., 2020; Pascale, 2020).

**Objectives:**

The study was held in 50 teachers, who worked remotely during the self-isolation due to COVID-19 pandemic, and aimed to estimate the relationship between psychological resources of self-regulation and signs of reduced professional burnout in teachers during the COVID-19 pandemic.

**Methods:**

. The assessment methods included: 1) Maddi’s “Hardiness survey”; 2) Hobfoll”s “SACS”; 3) Maslach’s “Burnout inventory”.

**Results:**

The results revealed that the teachers with high and medium burnout differ in terms of engagement as a component of hardiness (p=0,002). The teachers with less pronounced burnout syndrome have more developed involvement, which means that these teachers enjoy their own activities and, perhaps, this is what becomes a psychological resource and allows to overcome emotional exhaustion. There is an inverse relationship between such a sign of professional burnout as emotional exhaustion and involvement as a component of hardiness (r=- 0,521, p=0).

**Conclusions:**

The results of the study can be applied to develop programs to improve the psychological well-being and performance of teachers working under stress due to COVID-19 pandemic.

**Disclosure:**

No significant relationships.

